# Simulated herbivory in chickpea causes rapid changes in defense pathways and hormonal transcription networks of JA/ethylene/GA/auxin within minutes of wounding

**DOI:** 10.1038/srep44729

**Published:** 2017-03-16

**Authors:** Saurabh Prakash Pandey, Shruti Srivastava, Ridhi Goel, Deepika Lakhwani, Priya Singh, Mehar Hasan Asif, Aniruddha P. Sane

**Affiliations:** 1 Plant Gene Expression Lab, CSIR-National Botanical Research Institute, Lucknow-226001, India; 2 Academy of Scientific and Innovative Research (AcSIR), Anusandhan Bhawan, Rafi Marg, New Delhi-110 001, India; 3 Dept of Bioinformatics CSIR-National Botanical Research Institute, Lucknow-226001, India

## Abstract

Chickpea (*C. arietinum L*.) is an important pulse crop in Asian and African countries that suffers significant yield losses due to attacks by insects like *H. armigera*. To obtain insights into early responses of chickpea to insect attack, a transcriptomic analysis of chickpea leaves just 20 minutes after simulated herbivory was performed, using oral secretions of *H. armigera* coupled with mechanical wounding. Expression profiles revealed differential regulation of 8.4% of the total leaf transcriptome with 1334 genes up-regulated and 501 down-regulated upon wounding at log_2_-fold change (|FC| ≤ −1 and ≥1) and FDR value ≤ 0.05. *In silico* analysis showed the activation of defenses through up-regulation of genes of the phenylpropanoid pathway, pathogenesis, oxidases and CYTP450 besides differential regulation of kinases, phosphatases and transcription factors of the WRKY, MYB, ERFs, bZIP families. A substantial change in the regulation of hormonal networks was observed with up-regulation of JA and ethylene pathways and suppression of growth associated hormone pathways like GA and auxin within 20 minutes of wounding. Secondary qPCR comparison of selected genes showed that oral secretions often increased differential expression relative to mechanical damage alone. The studies provide new insights into early wound responses in chickpea.

Plants are the primary source of food for non-photosynthetic organisms and are constantly attacked and fed upon by microorganisms and insect/animal herbivores. To withstand these attacks, plants have evolved sophisticated defense mechanisms that include preformed structures like trichomes, spines, thorns and chemicals like antinutritional compounds, toxins and secondary metabolites^1^,^2^. Plants also possess inducible defenses that reduce the performance of attacking insects through synthesis of defense chemicals, protease inhibitors, chitinases and polyphenol oxidases^1^,^2^. These defenses are usually triggered by the mechanical wounding caused by the insects and the elicitors present in their oral secretions^3^,^4^,^5^,^6^.

From an agricultural perspective, the damage by insects causes great losses, being anywhere from 18–50% depending on the season. Pesticides, although effective, are harmful to the environment. To formulate better strategies against these pests a detailed analysis of the insect-plant interactions at various levels is needed. These include knowledge of insect-crop specific responses, genes that trigger/regulate these defenses, the timing of their expression and the mechanism of their action.

Large scale transcriptional maps in response to wounding and herbivory by different chewing, piercing and sucking pests have been generated in model plants such *Arabidopsis thaliana*^7^,^8^,^9^,^10^ and *Nicotiana attenuata*^11^,^12^ and in crops of importance like tomato^4^,^13^,^14^, *Citrus*^15^, cotton^16^,^17^, cucumber^18^, poplar and spruce^19^,^20^,^21^,^22^. These studies, on time scales from minutes to hours (mechanical wounding) and from hours to days (insect herbivory), show that plants respond with a complex transcriptional change in various phytohormone pathways, secondary metabolite pathways (phenylpropanoid and glucosinolate) and genes related to oxidative stresses and other regulatory genes^4^,^7^,^8^,^10^,^16^,^20^,^21^,^23^,^24^,^25^,^26^.

Earlier studies showed a considerable overlap in differentially expressed genes after various stresses such as wounding, herbivory or pathogen attack^8^,^21^. Subsequent studies have shown that the response also depends on the attackers, their feeding guides and may be species specific^24^. For instance, aphids and caterpillars elicit different responses with only a 10% overlap in up-regulated and 8% in down-regulated genes^10^. Two different caterpillars shared only 21% and 12% of overlap amongst up- and down-regulated genes. On the other hand, two different plants of the Solanaceous group responded to an attack of the same Lepidopteran herbivore by a species-specific differential gene expression pattern^27^.

Besides the insect and plant, the response is also dependent upon the duration after wounding with early responses being markedly different from late responses. For example, the expression in 30 min and 6 h time points^8^ or in 6 h and 24 h time points^10^ show little overlap. Most studies on insect wounding have been performed at time points of a few to several hours or days. The changes occurring within minutes of insect wounding, although important, have been far less characterized. These are important to understand how early wound signals are transmitted and to isolate early wound responsive promoters that could be more effective in targeting insect larvae within minutes of attack than conventionally used constitutive promoters for expression for insecticidal genes.

Chickpea (*C. arietinum*), is an important pulse crop that is subject to damage and yield losses due to insects like *H. armigera*^28^. The control of chickpea losses through genetic manipulation require an understanding of molecular events that lead to activation or repression of wound responsive genes after insect wounding. In the present study, we have investigated early wound responses in chickpea leaves within 20 minutes of simulated herbivory through an Illumina based transcriptome analysis. We show the activation of pathways related to defense and hormones like jasmonic acid (JA) and ethylene and simultaneous suppression of growth related hormones of gibberelic acid (GA) and auxin within minutes of wounding. The study represents an important resource for understanding wound responses in legumes for use in improvement of several crops.

## Results

### Illumina based sequencing and assembly of RNA from wounded chickpea leaves

To assess the early transcriptional responses of chickpea towards wounding, RNA from unwounded and 20 min wounded leaf tissue was used to generate RNA seq libraries for deep sequencing on an Illumina HiSeq 2000 platform. To mimic insect feeding, oral secretions from *Helicoverpa armigera* caterpillars were spread over the leaf surface just prior to wounding. Biological triplicates for wounded and unwounded leaves were included. On an average, 85% of total data from paired end sequencing (101 bp) passed > = 30 Phred score (Figure S1). Of the total clean reads obtained following initial quality filtering, 94% from control and wounded libraries could be completely mapped to the chickpea genome using TopHat software (Table S1).

Scatter plots of comparisons of biological replicates showed very little variation in expression between the biological triplicates of each sample in contrast to the comparison between wounded and unwounded samples (Figure S2A and B) indicating that the experimental data set was highly reproducible. Having established reproducibility, the samples were used for further analysis.

Cuffdiff program of Cufflinks package (version 2.2.1) was used to assemble the transcripts and estimate their abundance in wounded and unwounded tissues. The uniquely mapped reads were estimated as Fragments Per Kilo base of transcript per Million mapped reads (FPKM) and the differentially expressed genes (DEGs) in wounding were defined as significantly up- or down-regulated based on a log_2_-fold change (|FC| ≤ −1 and ≥1) with FDR value ≤ 0.05. Others with all FDR value having (|log_2_FC| ≥ 0.5 and ≤−0.5) were considered as differentially (up or down) regulated.

**Figure 1:**
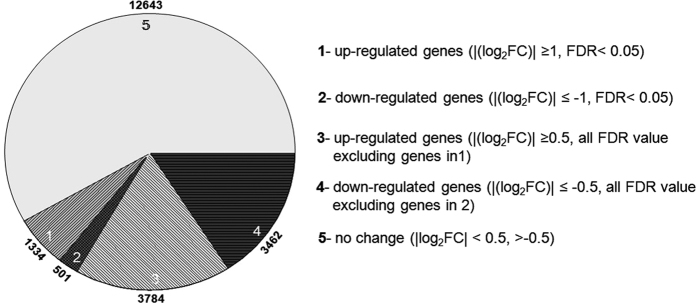
Overview of genes expressed in the 20 minute wounded leaf transcriptome following a comparison with unwounded leaves. Transcripts that satisfied the conditions of “FDR < 0.05” and “(Log_2_ |FC| ≤ −1 and ≥1)” were considered significantly differentially regulated genes (1 and 2). Others with all FDR value having (|Log_2_FC| ≤ −0.5 and ≥0.5) were considered as differentially up- or down-regulated (3 and 4 respectively). Transcripts that differed by (|log_2_FC| < 0.50 and >−0.5) were assumed to not change in expression level (5).

Following assembly, a total of 21724 genes out of 28,269 could be identified as expressed in the chickpea leaf transcriptome. Of these, a total of 1835 genes were significantly differentially regulated at FDR < 0.05 and fold change (≥1 and ≤−1) upon wounding. A total of 1334 were up-regulated and 501 down-regulated (Fig. 1), indicating that a larger number was activated upon wounding.

Next, a KEGG (Kyoto Encyclopedia of Genes and Genomes) analysis was performed with all 1835 DEGs using CAM Ids and the genes classified into 123 KEGG pathways. Amongst the top 15 KEGG pathways (Figure S3) were “metabolic pathways” (cam01100, 548 transcripts), “biosynthesis of secondary metabolites” (cam01110, 219 transcripts), “ribosome” (cam3010, 104 transcripts), “plant hormone signal transduction” (cam04075, 75 transcripts), “starch and sucrose metabolism” (cam00500, 73 transcripts) and “plant-pathogen interaction” (cam04626, 53 transcripts).

**Figure 2:**
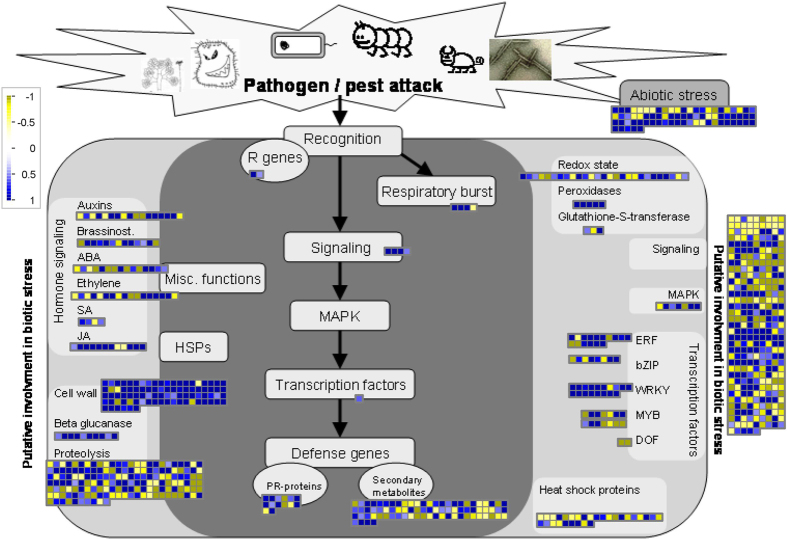
Overview of different groups and pathways regulated in chickpea after mechanical wounding by MapMan. All the genes showing Log_2_|FC| (≤−0.5 and ≥0.5) in expression (wounded vs. unwounded) were analyzed by MapMan software. Blue and yellow squares represent up- and down-regulated genes respectively. The colour saturation indicates Log_2_|FC| > 1 and <−1.

**Figure 3:**
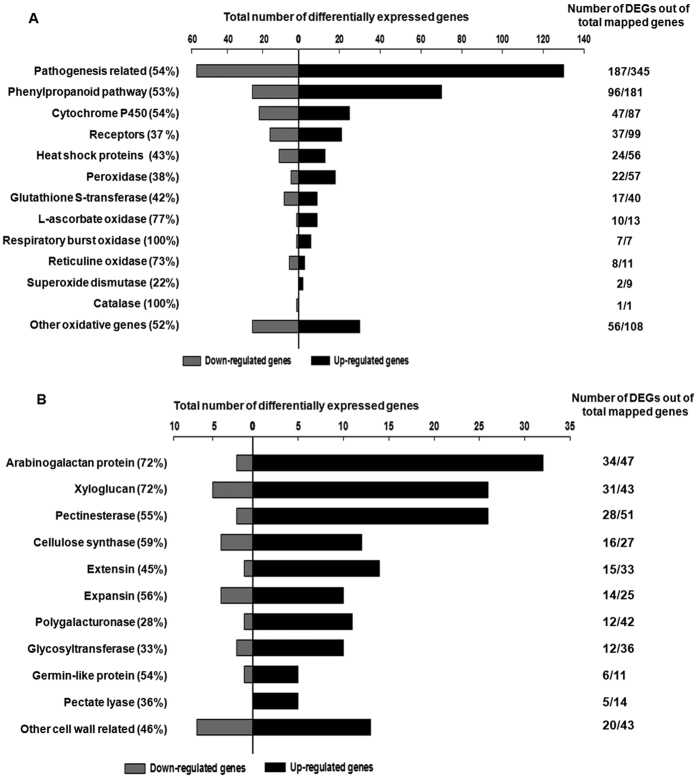
Functional cataloguing showing the proportion of differential wound-responsive genes (Log_2_|FC| (≤−0.5 and ≥0.5) associated with defense response (**A**) Pathogenesis, phenyl propanoid pathway, cytochrome P450, heat shock proteins and oxidative stress, receptors (**B**) Cell wall modification related genes. The differential genes belonging to each group were marked as up or down-regulated upon comparison with control. The numbers on the right indicate the DEGs as a fraction of the total genes in that group that were mapped on the chickpea genome in the wound transcriptome while the percentage of DEGs are shown on the left.

A MapMan tool^29^ analysis was next performed on all DEGs to identify important biological pathways affected during wounding. The most prominently affected genes belonged to the biotic/abiotic stress groups, secondary metabolite synthesis, cell wall modification, proteolysis, redox regulation, hormone signaling and transcription factor families (Fig. 2). Within biotic stress, those encoding pathogenesis related proteins, oxidative burst and the phenyl propanoid pathway proteins were abundant. Pathogenesis related genes formed the largest group responding to the early wound signal with 187 genes (Fig. 3A; Tables S2 and S3). These included those encoding proteinase inhibitors (PINs), endochitinase PR4, chitinase-like, thaumatin-like proteins, NPR-like homologues, disease resistant proteins and receptor genes with similarity to those encoding toll/interleukin-1 like receptors, systemin receptors and glutamate receptors, involved in recognition of pathogens, pests and damage associated molecular patterns (Fig. 3A and Table S3). Almost two thirds of these were up-regulated indicating the activation of defense responses upon wounding.

Several genes involved in oxidative burst (an essential component of the defense response) were also differentially regulated with more than 80% (of 123) being highly up-regulated. These included genes encoding enzymes involved in hydrogen peroxide (H_2_O_2_) production/processing, glutathione S transferase, peroxidase, ascorbate oxidase, respiratory burst oxidase protein D (RbOPD), superoxide dismutase and other oxidases (Fig. 3A; Table S2).

The phenylpropanoid pathway, an important defense related pathway, contained at least 96 DEGs (Fig. 3A) of which about 72%, were up-regulated within 20 minutes. These included the gene encoding phenylalanine ammonia-lyase (PAL) – a key regulator and the branch point enzyme between primary and secondary metabolism and other genes such as 4-coumerate: CoA ligase 5, cinnamoyl-CoA reductase, caffeoylshikimate esterase, caffeoyl-CoA 3-O-methyltransferase, 4-hydroxyphenylpyruvate dioxygenase (Table S2).

The cytochrome P450 oxidase family (CYP450s) regulates metabolic processes of the phenylpropanoid, alkaloid and terpenoid pathways. A total of 47 CYP genes were detected in the wound transcriptome of which 25 and 22 were up- and down-regulated, respectively (Fig. 3A and Table S2). Homologues of some of these such as the Arabidopsis *CYP77A1* are associated with the phenylpropanoid pathway while others like *CYP94, CYP86, CYP84*, are associated with the fatty acid ω-hydroxylation pathway involved in regulation of JA-Ile turnover^30^,^31^,^32^.

HSPs, have also been reported to be differentially regulated upon wounding and plant defense^33^,^34^,^35^. Of the 24 HSPs detected, 13 were up-regulated and 11 were down-regulated upon wounding (Fig. 3A; Table S2).

Genes involved in biosynthesis and modifications of cell wall components such as pectin, cellulose, hemicellulose and proteoglycans were highly represented with 193 genes in the transcriptome. Most of these (almost 80%) encoding enzymes such as xyloglucan endotransglycosylase/hydrolases (XTH), pectin esterase, cellulose synthase (CESA), expansin, pectate lyase, polygalacturonase and arabinogalacton proteins were prominently up-regulated upon wounding (Fig. 3B; Table S2).

Genes related to sugar metabolism did not appear to undergo much change.

### Expression profiles of genes related to transcriptional regulation

**Figure 4:**
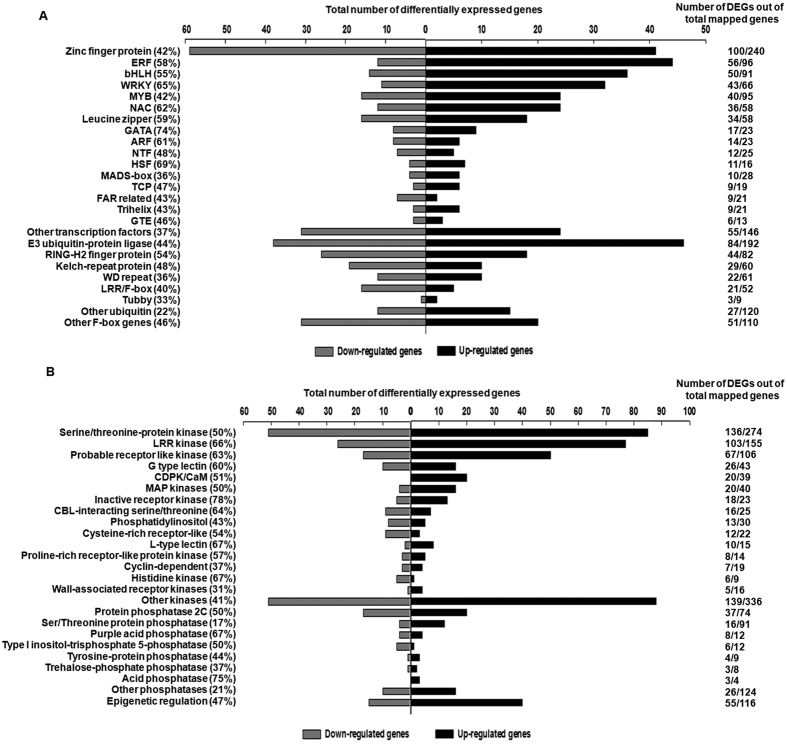
Functional cataloguing showing the proportion of differential wound-responsive genes [Log_2_|FC| (≤−0.5 and ≥0.5)], associated with signaling and regulatory proteins. (**A**) Transcription factors, F-box/proteolysis components (**B**) Various kinases, phosphatases and epigenetic regulators. The numbers on the right indicate the DEGs as a fraction of the total genes in that group that were mapped on the chickpea genome in the wound transcriptome while the percentage of DEGs are shown on the left.

The large and rapid change in the wound transcriptome within 20 minutes of wounding would require the action of several transcription factors that target different pathways for an effective wound response. Indeed, with 511 DEGs (293 up and 218 down), this was one of the largest groups (Table S3). Genes encoding Zn finger domain proteins formed the largest sub-class with 100 DEGs (Fig. 4A). The majority, comprising ~60%, were down-regulated upon wounding. Other major groups included bHLH domain genes (36 up- and 14 down-regulated), MYBs (24 up-, 16 down-regulated), ERFs (44 up-, 12 down-regulated), WRKYs (32 up-, 11 down-regulated), NACs (24 up-, 11 down-regulated), and leucine zipper protein genes (18 up-, 16 down-regulated; Fig. 4A; Table S3). HSFs, GATA, ARFs and MADS, were also differentially regulated as shown in Fig. 4A.

A great deal of regulation is also brought about by proteins that control turnover of key regulatory proteins in important cellular processes governing cell cycle, stress and signal transduction pathways^36^. A large group of 281 DEGs encoded E3 ubiquitin ligases and F-box proteins with additional domains such as RING-H2 finger, WD repeat, Tubby, LRR, Kelch etc (Fig. 4A). A greater proportion of these (155/281) were down-regulated upon wounding.

Phosphorylation/dephosphorylation play an important role in perceiving and rapidly responding to stress signals. These accounted for 606 DEGs in the chickpea wound transcriptome encoding different types of kinases (Fig. 4B, Table S3) of which 402 were up-regulated while 204 were down-regulated. The majority encoded serine threonine protein kinases (85 up-, 51 down- regulated), LRR kinases (77 up- and 26 down-regulated) and receptor-like kinases (50 up, 17 down). Calcium dependent protein kinases (CDPKs), mitogen-activated protein kinases (MAPKs), G-type lectin kinases and CBL interacting protein kinases were also abundant. Interestingly, more than 80% of the differentially regulated MAPKs and all CDPKs were up-regulated while most of the phosphatidyl inositol kinases (8 out of 13), cysteine rich receptor kinases (9 out of 12), histidine kinases (5 out of 6) and some CBL interacting protein kinases (9 out of 16), were down-regulated.

In contrast to kinases, phosphatases accounted for a much smaller proportion of the DEGs with 103 genes of which 61 were up-regulated and 42 were down-regulated. The majority of the phosphatase genes belonged to the PP2C and PP2A groups. Of these, most PP2A members were up-regulated while the PP2C class showed an even proportion of up- and down-regulated genes (Fig. 4B; Table S3).

In addition, several genes encoding histone proteins and histone modification enzymes were differentially regulated with most histone protein genes being up-regulated. (Table S3).

### Expression profiles of phytohormone related genes

**Figure 5:**
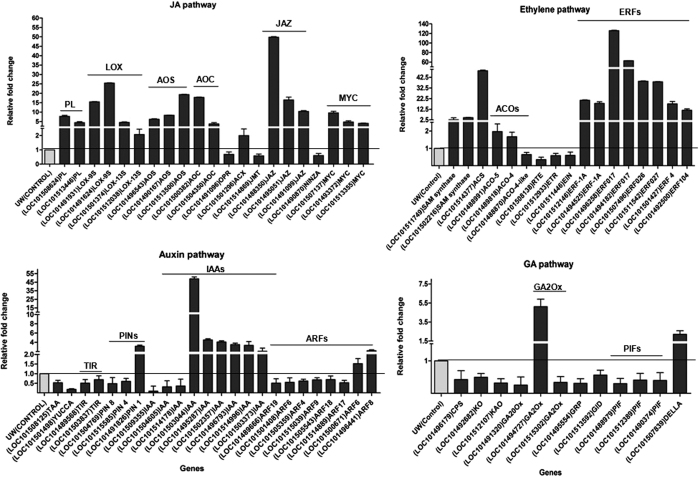
Expression profile of differential wound-responsive genes related to JA, ethylene, GA and auxin pathways. Bars represent the relative fold expression change after simulated herbivory as calculated from transcriptome data using the expression in all three biological replicates (Log_2_ |FC| ≤ −1 and ≥1, FDR < 0.05). Expression in respective controls was taken as one and shown as a black line across genes for comparison. Data is represented as the mean of ±SE of biological replicates.

Phytohormones regulate almost every aspect of plant growth in response to developmental cues and biotic/abiotic stresses. They are also known to be regulated in response to wounding and insect attack. To examine how wound signaling in chickpea affected phytohormone pathways, genes encoding biosynthesis/signaling components of JA, salicylic acid (SA) and ethylene (ET), that are important in regulating defense responses against biotic threats^37^ as well as other phytohormones, namely auxin (AUX), abscisic acid (ABA), GA, cytokinin (CYT) and brassinosteroid (BS) were analyzed. The analysis indicated a prominent up-regulation of the JA and ethylene pathways both at the level of biosynthesis and response/signaling with an over-representation in the wound-responsive transcriptome (Fig. 5). For the JA pathway, these included up-regulation of homologues of several phospholipase genes, lipoxygenase genes encoding 9-LOX and 13-LOX and those encoding allene oxide synthases (AOSs), allene oxide cyclases (AOCs) and homologues of two *JASMONIC ACID RESISTANCE* genes (JAR1s) all of which are involved in JA biosynthesis (Fig. 5). Three *MYC* homologues that are known to govern JA responses in Arabidopsis and several jasmonate *ZIM (JAZ*) homologues were also up-regulated. Within the ethylene pathway the key genes involved in biosynthesis (SAM synthases; ACC synthetases and ACC oxidases) were up-regulated. A large number of the ethylene responsive factor (ERFs) were also up-regulated as already discussed above while an F-box gene encoding EBF (a negative regulator of EIN3) was down-regulated (Fig. 5). In contrast, genes associated with the GA pathway were prominently down-regulated by wounding. These included genes encoding homologues of ent-copalyl diphosphate synthase (CPS), ent-kaurene oxidase (KO), ent-kaurenoic acid oxidase (KAO) and several GA20 oxidases involved in biosynthesis. GA2 oxidases, that inactivate GAs, were up-regulated. PIF genes, involved in growth and cell elongation along with GA, were also suppressed while transcription of the GA suppressor DELLA was increased (Fig. 5). The auxin pathway was also down-regulated with homologues of *TIR, YUCCA, PIN* and several *ARF* genes being down-regulated while several AUX/IAA genes were up-regulated (Fig. 5).

**Figure 6:**
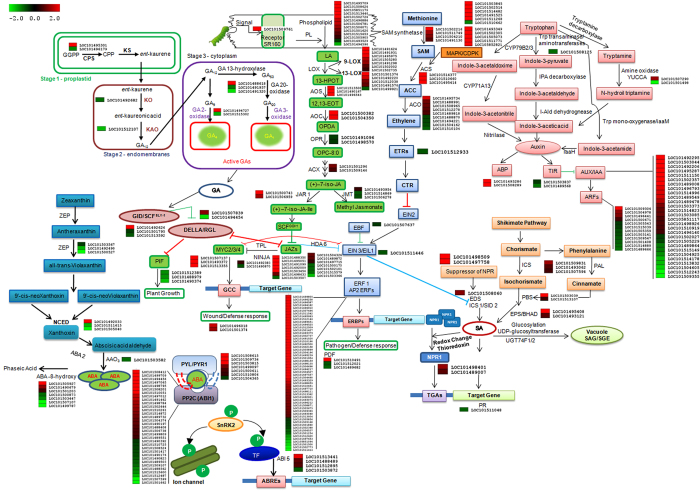
Overview of hormonal pathways and their cross talk occur at transcript level after simulated herbivory. Red and green colour squares represent up- and down-regulated genes [Log_2_|FC| (≤−0.5 and ≥0.5)] respectively. The colour saturation indicates log_2_ fold change between −2 and 2.

The ABA pathway showed a mixed response. While some of the biosynthesis related genes such as ZEPs and one 9-cis-epoxycarotenoid dioxygenase (NCED) were up-regulated others such as *ABA2* were down regulated. The gene encoding ABA 8-hydroxylase (that degrades ABA) was also down-regulated. This, along with the up-regulation of a few *PYL* like receptor genes (in particular *PYL4* like) were suggestive of an increase in ABA response and signaling. Genes associated with SA, cytokinin and brassinosteroid pathways were not over-represented among the differentially expressed genes (Table S4). An overview of the changes in various hormonal pathways is shown in Fig. 6.

### Expression analysis of top hundred regulated genes

**Figure 7:**
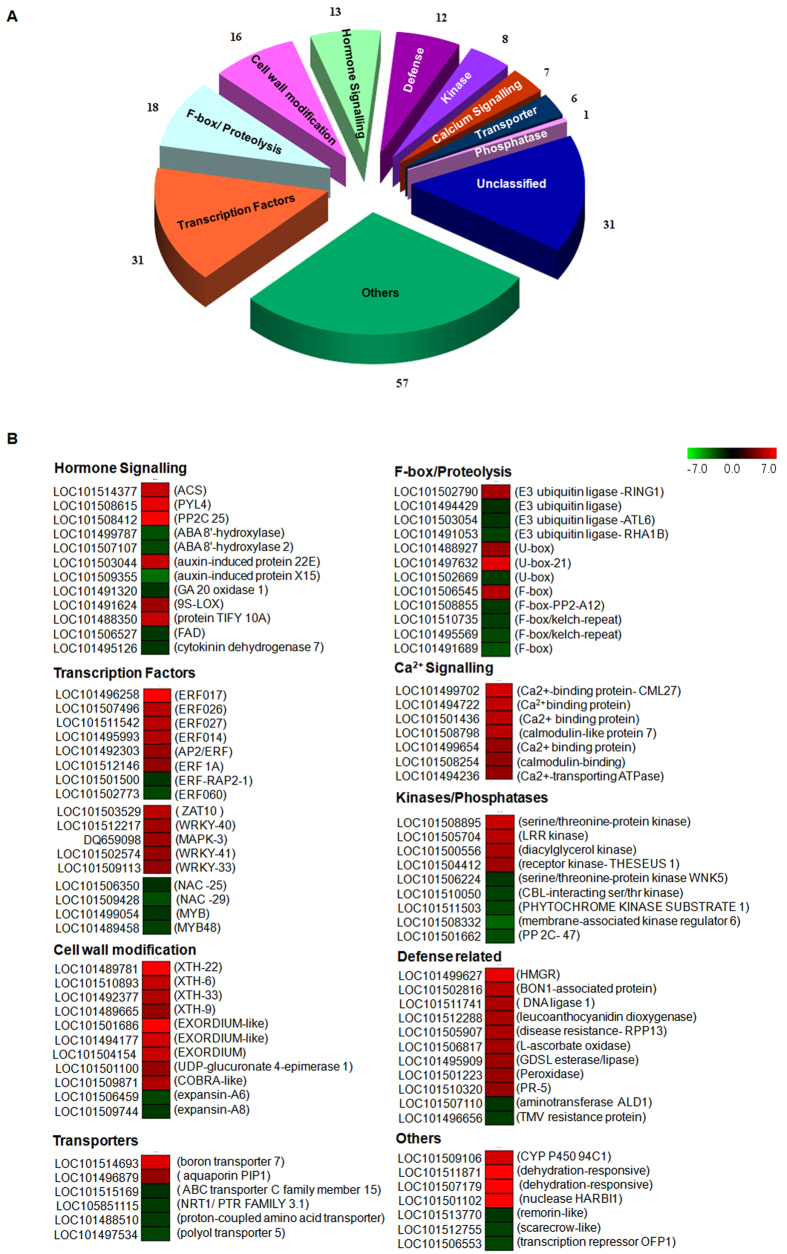
Proportion and expression pattern of various groups in the top 100 up and down regulated genes of the 20 minute wounded chickpea leaf transcriptome. (**A**) Pie diagram showing the various groups to which the top 100 up- and down-regulated genes in chickpea wound transcriptome belong to. (**B**) Heatmap of the top 100 differentially wound regulated genes in different categories. The red and green colours indicate the up and down regulation of genes, shown with + and – signs respectively in the colour bar at the top of the heat map.

In order to identify genes undergoing the maximum transcriptional changes within minutes of wounding in chickpea, an expression analysis of top hundred up- and down-regulated genes was performed (Fig. 7A). Of the dominantly represented groups in Fig. 7A, several ERFs, WRKYs, cell wall modifying genes, calcium binding protein genes and defense related genes were up-regulated (Fig. 7B). The groups containing F-box genes and the NAC/MYB transcription factors were mostly down-regulated while those belonging to kinases and hormone signaling showed both up- and down-regulated genes.

### Salivary elicitors exert an added regulation over mechanical wounding in the wound transcriptome

**Figure 8:**
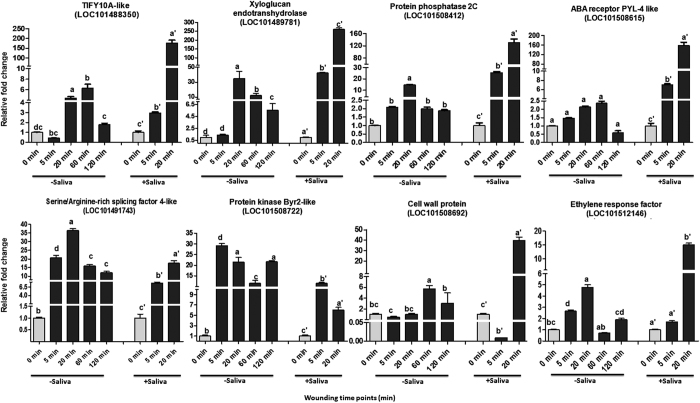
Comparative Real Time PCR validations of selected genes in mechanically wounded and simulated herbivory wounded chickpea leaves. Expression was studied at 0, 5, 20, 60 and 120 min after mechanical wounding and compared to 0, 5 and 20 min patterns of simulated herbivory wounding (saliva treated) with 0 min being the unwounded control in both set of experiments. The real time expression data was normalized using two validated reference genes *EF1alpha* and *HSP*-*90*. Error bars represent ±SE of three biological replicates. Expression values were analyzed by one-way ANOVA and compared using Duncan’s Multiple Range Test (DMRT). Values on the bar carrying different letters are significantly different (α = 0.05).

Finally, to get an insight into expression dynamics of some of the wound up-regulated genes during early phases of wound response, the expression of eight differential genes (selected randomly, Supplementary Table S5) was studied at time points ranging from 5 min to 120 minutes with a comparison between mechanical wounding and simulated herbivory (mechanical + saliva treated samples) at 5 and 20 minutes. As shown (Fig. 8) the expression of most genes was up-regulated upon mechanical wounding within 5–20 minutes. In most cases the transcript level of genes peaked at 20 minutes and came down by 2 h. Interestingly, the expression of the genes at corresponding time points was higher in simulated herbivory for six of the eight genes studied indicating that salivary factors probably acted as elicitors in activating their expression. In two cases, however (LOC101491743 and LOC101508722), the expression upon simulated herbivory was lower than that of mechanical wounding.

## Discussion

In the race for survival, insects and plants have constantly evolved strategies for their protection leading to a complex network of insect-plant interactions. Understanding these is essential to develop means for protection of crops of importance against major insects. *Cicer arietinum* L. is an economically important legume that is subject to losses by chewing pests like *Helicoverpa armigera*^28^. In this study, we have attempted to understand some of the earliest responses of chickpea leaves to simulated herbivory using oral secretions of *H. armigera* followed by mechanical wounding, so as to get a more realistic idea of early insect wound responsive genes. Compared to previous studies where response times of a few hours or days were tested^4^,^8^,^10^,^20^,^23^,^26^, transcriptional responses occurring within minutes of insect wounding have been far less characterized due to practical problems getting enough wounded tissue within minutes using live insects.

A total of 1835 differentially expressed genes were identified (at log_2_FC ≥ 1, ≤−1, FDR < 0.05) of which 1334 were induced and 501 genes were repressed significantly. Even this early time frame could bring about a significant change in almost 8.4% of the assembled leaf transcriptome (1835 DEGs out of 21724 expressed genes) confirming that wounding constitutes a major stress signal^7^,^8^,^24^. The expression of reference genes identified by Garg *et al*.^38^ for chickpea was not found to differ even upon wounding, indicating their utility as reference for wound related studies (Figure S4).

The major functional categories of the DEGs obtained using KEGG and MAPMAN pathway analysis corresponded to those involved in activating defense responses, oxidative stress, pathogenesis related genes, secondary metabolite production through the phenylpropanoid pathway and cell wall modification (Fig. 3A and B) some of which were also identified previously^21^. Genes in these pathways are activated by wounding and insect herbivory in Arabidopsis^7^,^8^,^23^, tomato^14^, *Nicotiana attenuata*^12^, *Barbarea vulgaris*^26^ and *Gossypium hirsutum*^16^. However, in contrast to results by Cheong *et al*.^8^ where most of these pathway genes were activated late at 6 h instead of 30 minutes, our results show up-regulation of these genes within 20 minutes.

Secondary metabolites like flavonoids and isoflavonoids from the phenylpropanoid pathway play a decisive role as chemical deterrents in resistance against pathogens and herbivore infestation^39^,^40^,^41^,^42^. In chickpea, the transcription of many genes in the phenyl propanoid pathway such as PAL, cinnamoyl-CoA reductase, caffeoyl-CoA 3-O-methyltransferase, chalcone-synthase (Table S2) increased within minutes of wounding suggesting an important role for flavonoid metabolism in the defense response of chickpea.

The analysis also confirmed that oxidative burst, an essential component of wound signaling, is regulated early upon wounding in chickpea. The accumulation of reactive oxygen species (ROS) is an early event occurring within minutes after wounding/herbivory^43^. H_2_O_2_ accumulation has been proposed as a local diffusible signal for activation of defense genes^2^,^44^. The up-regulation of respiratory burst oxidase homologues (RBOH) and other genes associated with ROS system such as ascorbate oxidases and peroxidases confirms their role in early defense response in chickpea^23^,^21^.

One of the primary findings of our studies has been the rapid transcriptional activation of the JA and ethylene pathways within 20 minutes of wounding. The octadecanoid JA biosynthesis pathway is known to have a central role in response to insect herbivory or mechanical damage in plants^43^. In Arabidopsis, JA governs 44% of the wound up-regulated genes and 46% of the down-regulated genes^45^. Besides JA, the ethylene pathway is also up-regulated upon wounding^8^,^23^. Consistent with these studies, the most dramatic response to wounding was up-regulation of JA and ethylene pathways (Figs 5 and 6) seen as an increase in biosynthesis as well as signaling associated RNAs. These included most JA biosynthesis genes such as various *LOX*s, *AOS*s, *AOC*s and the *MYC* homologues that activate JA responsive genes^2^,^16^. Transcription of many *JAZ* inhibitor genes was also up-regulated possibly as negative feedback regulation. The up-regulation of the JA pathway supports an important role for JA in chickpea upon wounding. The ethylene pathway was also rapidly up-regulated with genes involved in biosynthesis (SAM synthetases, ACS and ACO) showing an increase of 1.6–2 folds as noted before for ACO^25^. Wound-mediated ethylene production has been shown to reduce susceptibility of JA to inhibition by SA thereby providing greater protection against insect herbivory^46^,^47^. A rapid induction of many ERFs was also observed with an overrepresentation in top 100 up-regulated group (Fig. 7B). ERFs have been reported to be rapidly up-regulated by wounding^48^,^49^ and also associated with integrating the JA-ethylene signal pathways as evident from studies on *ERF1* and *ORA59*^50^,^51^. The rapid induction of the JA/ethylene pathways suggests a crosstalk between wounding, JA and ethylene right from the level of transcriptional regulation in chickpea.

The activation of defense pathways upon insect herbivory requires diversion of carbon supply from growth related activities towards defense. These stresses often suppress growth pathways while the plant combats the stress^52^. How early these decisions are made is not clear. Our studies show that wounding effects a suppression of the growth pathways governed by GA and to some extent auxin within 20 minutes of wounding in chickpea. This was apparent from down-regulation of homologues of several GA biosynthesis (*CPS, KO, KAO, GA20OX*) genes and the GA receptor homologue *GID* but up-regulation of GA catabolism gene (*GA2OX*). Simultaneously, up-regulation of the GA suppressor *DELLA* was also observed (Fig. 5). GA controls growth by regulating the degradation of growth repressing DELLA proteins^53^, while JA antagonizes GA action by inducing *DELLA* via MYC2 in a COI/MYC2 dependent manner to enhance the JA response^52^,^54^. DELLA proteins bind to JAZ1, thereby suppressing its interactions with MYC2 and allowing MYC2 to activate JA-responsive target genes^54^,^55^. In chickpea, the up-regulation of DELLA and JA pathways (including MYC2 homologues) and the simultaneous suppression of GA pathway suggests similarities between Arabidopsis and chickpea in the JA-DELLA-MYC2-GA interactions although these will require further confirmation. Auxin also activates growth through activation of cell division and mitosis^56^ and its action in cell expansion is suppressed by JA and ethylene in *N. attenuata*^57^. In chickpea too, the auxin pathway seems to be suppressed upon wounding through suppression of *YUCCA, TIR, PIN* and several *ARF* genes and a simultaneous transcriptional activation of some *AUX*/*IAA* genes that function as repressors. The possible increase in production of flavonoids through up-regulation of the phenylpropanoid pathway may also inhibit auxin action since many of these compounds function as auxin suppressors^58^. Auxin levels decrease in tobacco and maize after wounding or herbivory^57^,^59^ although a recent report suggests activation of auxin levels in *N. attenuata* in response to attack by *M. sexta*^60^. Auxin related genes were down-regulated in Arabidopsis after 30 min and 6 h of wounding^8^. Also, external application of auxin suppressed wound responses, jasmonate production^61^ and proteinase inhibitor expression^59^,^62^. The opposite relationship between auxin and jasmonate pathways upon wounding seems to be conserved in chickpea. What is interesting is that the decisions to suppress the growth pathways mediated by GA and auxin are taken within 20 minutes of wounding and occur at the level of hormone biosynthesis, signaling and response.

The transcriptional activation of the defense and hormonal pathway genes described above requires action of several transcription factors and regulatory genes encoding kinases, phosphatases, receptors and various F-box proteins. This was also evident in the chickpea wound transcriptome where several TFs of the MYB, WRKY, bHLH families (besides the ERF and MYC family described above) were up-regulated within 20 minutes of simulated herbivory (Fig. 4A). The MYB family transcription factors play an important role in defense responses against wounding^8^, herbivory^26^ and pathogens^63^. They also control multiple steps of phenylpropanoid pathway in defense^64^. Interestingly, wounding in chickpea up-regulated both, the MYB family and the phenylpropanoid pathway (Tables S2 and S3) suggesting similarities in regulation of the phenylpropanoid pathway by MYBs in chickpea too. WRKY genes are also activated early in wounding^8^ besides being associated with responses to pathogen attack and SA^65^. It is likely that up-regulation of WRKYs may regulate defenses against herbivory besides suppressing attack by pathogens that gain entry through wounding.

Kinases and phosphatases are important regulatory enzymes in stress signaling and wounding with the MAPK pathway being an important player in triggering defense responses^66^. In tomato, perception of wound-induced systemin by its receptor SR160 triggers MPK1 and MPK2 activation and JA biosynthesis^67^. The MEKK1-MEK1/MEK2-MPK4 module is also active during wound signaling in Arabidopsis^68^. MAPKs such as WIPK (a wound induced MAP kinase from tobacco) and SIPK (a salicylate induced MAPK) are induced by wounding in tobacco, activated by NaMEK2/NtMEK2 upon herbivory and wounding^69^ and trigger wound induced JA accumulation and SA suppression^70^. Silencing these genes reduces JA suggesting their necessity for JA biosynthesis. Interestingly, homologues of WIPK (DQ659098) and SIPK (LOC101496681) were up-regulated within 20 minutes of wounding in chickpea, showing that regulation of the MAPK pathway may be as important for wound signaling in chickpea as in other plants and is currently under study in our lab.

Amongst other regulators, several receptor-like kinase genes were differentially regulated upon wounding as reported previously in herbivory^71^. In tomato, the systemin-receptor complex induces the activity of phospholipases which in turn make linoleic and linolenic acids available for JA biosynthesis^39^,^72^. In chickpea too, the up-regulation of SR-160 LRK and phospholipase genes hint towards a similar mode of action. Interestingly, the group encoding all calcium-dependent protein kinases was up-regulated. A simultaneous increase in expression of Ca^+2^ signaling genes was also found in the top 100 up-regulated section (Fig. 7B). Calcium ions (Ca^+2^) serve as secondary messengers mediating wound signaling within seconds of herbivore attack in plants^43^ and Ca^+2^ sensors activate downstream defense signaling cascades through calcium-dependent protein kinases for a few hours after damage^73^,^74^,^75^.

Herbivore damage requires repair of the cell wall to prevent pathogen entry^76^. Accordingly, most cell wall repair genes have been reported to be up-regulated upon wounding^8^,^77^. Expectedly, genes encoding cellulose synthase and XTHs, extensins, expansins and arabinogalacton proteins were rapidly up-regulated in chickpea. However, in contrast to Arabidopsis where most of these were altered late (at 6 h instead of 30 min), in chickpea these were activated within 20 minutes.

The entire transcriptome represents early changes occurring within 20 minutes of simulated herbivory that includes both, mechanical wounding and salivary factors. Each of these factors seems to contribute differentially as seen by the comparative time course expression dynamics of a few selected genes (Fig. 8). In at least 6 of the 8 genes studied, salivary factors seem to cause a greater induction of transcription compared to only mechanical wounding while in two genes (LOC101491743 and LOC101508722) there was a reduction in expression. That plants respond to different insects specifically has been documented in several elegant studies^8^,^10^,^27^,^78^. These studies show that specific salivary factors differently influence plant responses by activation or suppression of wound responsive expression^3^,^4^,^79^,^80^,^81^,^82^ with further complexities occurring due to duration and scale of wounding^5^. The expression dynamics of the eight genes also shows that most of these are only transiently up-regulated between 5–60 minutes before coming down. This is important since it means that many of these genes (and therefore their functions) could be missed out in studies where time points of a few to several hours are studied.

In conclusion, we provide a comprehensive transcriptomic analysis of changes in chickpea that show rapid regulation and rewiring of various hormonal networks leading to activation of JA and ethylene pathways and a simultaneous suppression of growth pathways governed by GA and auxin even within 20 minutes of simulated herbivory by *H. armigera*. These might be important for activating primary and secondary defenses against insect herbivores.

## Materials and Methods

### Plant material, growth condition and treatment

Chickpea seeds (*C. arietinum* var. Pusa 362) procured from the Indian Agricultural Research Institute, New Delhi, India, were grown in the fields of CSIR-NBRI in winters from November to March. Newly emerged and fully expanded bi-pinnate leaves of eight week old chickpea plants were wounded with a pair of pointed forceps by rapid and repeated pricking while still on the plant. Immediately prior to wounding, the tissue was exposed to insect saliva (simulated herbivory) obtained from the oral secretions of *H. armigera* (spread over the tissue with a soft brush). The wounded tissue was kept for 5 and 20 min on the plant, excised, frozen in liquid nitrogen, followed by RNA isolation. The 20 min RNA was used for Illumina sequencing and for validation of gene expression. For mechanical wounding, RNA was isolated without application of saliva to leaves at time points of 5, 20, 60 and 120 minutes after wounding.

### RNA extraction and quality controls

Total RNA was extracted from three parallel independent biological sets of unwounded and 20 min wounded leaves using plant total RNA isolation kit (Sigma) according to manufacturer’s instructions. The concentration of RNA samples was determined using a Nanodrop ND-1000 spectrophotometer (Nanodrop technologies, Wilmington, DE, USA). The quality and quantity of RNA was again checked using Agilent 2100 Bioanalyzer RNA chip (Agilent Technologies Inc., Santa Clara, CA). The integrity of RNA was assessed by electrophoresis on a 1.2% agarose gel in 0.5X TBE. Only the RNA samples with 260/280 ratios from 1.8 to 1.9, 260/230 ratios from 2.0 to 2.5 and RIN (RNA integrity number) more than 6.0 were used for the analysis. This RNA was used for Illumina sequencing and gene expression analysis.

### Illumina sequencing and data processing

The cDNA libraries were generated using mRNA assay for sequencing on Illumina HiSeq 2000 sequencing platform at Scigenome (Kochi, Kerala). Paired-end cDNA library was generated from all samples and sequencing was performed to generate ~100 bp paired-end reads. The initial filtering step (for removal of contaminating rRNAs, tRNAs, adapter sequences, low quality sequences and ambiguous bases) was performed using NGSQCTOOLKIT (http://www.nipgr.res.in/ngsqctoolkit.html) software^83^, in-house Perl scripts and Picard tools (version 1.100). NGSQCTOOLKIT software was used for filtering of high quality reads based on quality score (Q > 30) so as to retain only high quality sequence for further analysis. Each library generated about ~85% high quality reads for analysis.

### Assembly and Gene Expression Analysis

The pre-processed reads were aligned to the reference chickpea genome and gene model downloaded from NCBI (ftp://ftp.ncbi.nlm.nih.gov/genomes/all/GCF_000331145. 1_ASM33114v1/GCF_000331145.1_ASM33114v1_genomic.fna.gz). The alignment was performed using TopHat program version 2.0.8 (http://ccb.jhu.edu/software/tophat/index.shtml) and Bowtie2 (version 2.2.6.0) with default parameters^84^. Only reads that mapped to the genome were used for further analysis. The number of uniquely mapped reads for each gene model in the chickpea genome were calculated^85^. The aligned reads were used for estimating expression of the genes and transcripts using Cufflinks program (version 2.0.2). DGS (differential gene set) were identified by parsing the alignment output files from TopHat and the resulting read counts were then normalized by FPKM to measure the gene expression level. Differential expression analysis was performed using Cuffdiff program (version 2.0.2)^75^ using Cufflink package (http:/ial/cole-trapnell-lab.github.io/cufflinks/manual/).

### Functional annotation

To retrieve the detailed Kyoto Encyclopedia of Genes and Genomes (KEGG) pathway IDs, all the wound responsive up- and down-regulated genes in chickpea having fold change (<−1 and >1) and FDR value < 0.05 were analyzed using the KEGG database (http://www.genome.jp/kegg/)^86^,^87^. A heat map was generated for the differentially regulated contigs using MeV (version 4.8.1). Additionally, all the differentially regulated genes were also functionally analyzed using the MapMan software, which is a user-driven tool that displays large genomic datasets onto diagrams of metabolic pathways or other processes such as biotic stress^29^. For functional cataloguing of different groups of genes (Figs 3 and 4) DEGs with (Log_2_|FC| (≤−0.5 and ≥0.5) were chosen and grouped under various categories. For analysis of hormonal networks (Fig. 5) DEGs with a fold change (<−1 and >1) and FDR value < 0.05 were used.

### Validation of mRNA-seq data using qRT-PCR

The cDNAs were generated from above RNA samples using the REVERTAID MMLV kit (Fermentas) and used as template for validation of expression analysis of genes obtained in Illumina sequencing by real time PCR. Real time PCR primers (Table S2) were designed using the software Primer Express 2.0 (PE Applied Biosystems, USA). To ensure that each pair of primers amplified only desired cDNA fragment, each pair was checked using the BLAST program in chickpea genomic sequence available in NCBI database. Real time PCR was performed in 20 μl for a set of selected genes listed in Table S6 using SYBR Green PCR Master Mix (ABI, USA) using the following cycle conditions: 94 °C for 2 min, followed by 30 cycles of 94 °C for 30 s, 60 °C for 30 s, and 72 °C for 30 s, and the final 5 min extension at 72 °C. The specificity of the amplicon formed at the end of PCR was analyzed by performing a melting curve analysis. The relative mRNA level of the individual genes in different RNA samples was normalized with respect to validated internal control genes EF1α and HSP90^38^ listed in Table S6. Reactions were run in three biological replicates and three technical replicates on an ABI Prism 7500 real time PCR machine (Applied Biosystems Inc, USA). The analyzed real time reaction data was the mean of biological and technical triplicates in real time reaction. Relative gene expression was calculated using 2^−ΔΔCT^ method^88^.

### Data access

The raw data was deposited in the NCBI sequence read archive under BioProject ID: PRJNA328302, having BioSamples ID:SAMN05369585(UW-1), SAMN05369586(UW-2), SAMN05369587(UW-3), SAMN05369588(W-1), SAMN05369589(W-2)- SAMN05369590(W-3), SRA Accession ID- SRP078184. Direct link to deposited data http://www.ncbi.nlm.nih.gov/sra/SRP078184.

## Additional Information

**How to cite this article:** Pandey, S. P. *et al*. Simulated herbivory in chickpea causes rapid changes in defense pathways and hormonal transcription networks of JA/ethylene/GA/auxin within minutes of wounding. *Sci. Rep.* **7**, 44729; doi: 10.1038/srep44729 (2017).

**Publisher's note:** Springer Nature remains neutral with regard to jurisdictional claims in published maps and institutional affiliations.

## Supplementary Material

Supplementary Figures and Tables
