# Supplementary material for: Simulated herbivory in chickpea causes rapid changes in defense pathways and hormonal transcription networks of JA/ethylene/GA/auxin within minutes of wounding
Source: Sci Rep. 2017 Mar 16;7:44729. doi: 10.1038/srep44729 (PMC5353604; doi:10.1038/srep44729)
Supplement: Supplementary Figures and Tables [file srep44729-s1.pdf]

Simulated herbivory in chickpea causes rapid changes in defense pathways and hormonal transcription networks of JA/ethylene/GA/auxin within minutes of wounding

Saurabh Prakah Pandey[1,3], Shruti Srivastava[1], Ridhi Goel[2,3], Deepika Lakhwani[2,3], Priya Singh[1,3], Mehar Hasan Asif[2,3], Aniruddha P. Sane[1,3*]

[1]Plant Gene Expression Lab and [2]Dept of Bioinformatics CSIR-National Botanical Research Institute, Lucknow-226001, INDIA, [3]Academy of Scientific and Innovative Research (AcSIR), Anusandhan Bhawan, Rafi Marg, New Delhi-110 001, India

*Corresponding author

Email addresses of the authors:

Saurabh Prakash Pandey: praksaurabh@gmail.com

Shruti Srivastava shrutibiotech07@gmail.com

Ridhi Goel: Ridhi.goel2003@gmail.com

Deepika Lakhwani: lakhwanideepika@gmail.com

Priya Singh: priya.willis@gmail.com

Mehar Hasan Asif: mh.asif@nbri.res.in, meharasif@hotmail.com

Aniruddha P. Sane: ap.sane@nbri.res.in, saneanil@rediffmail.com

Supplementary figures and tables

Figure S1: Quality distribution plot of R_1 and R_2 reads showing average base quality (Phred score ≥30) in all biological replicates of the wounded and unwounded chickpea leaf transcriptome.

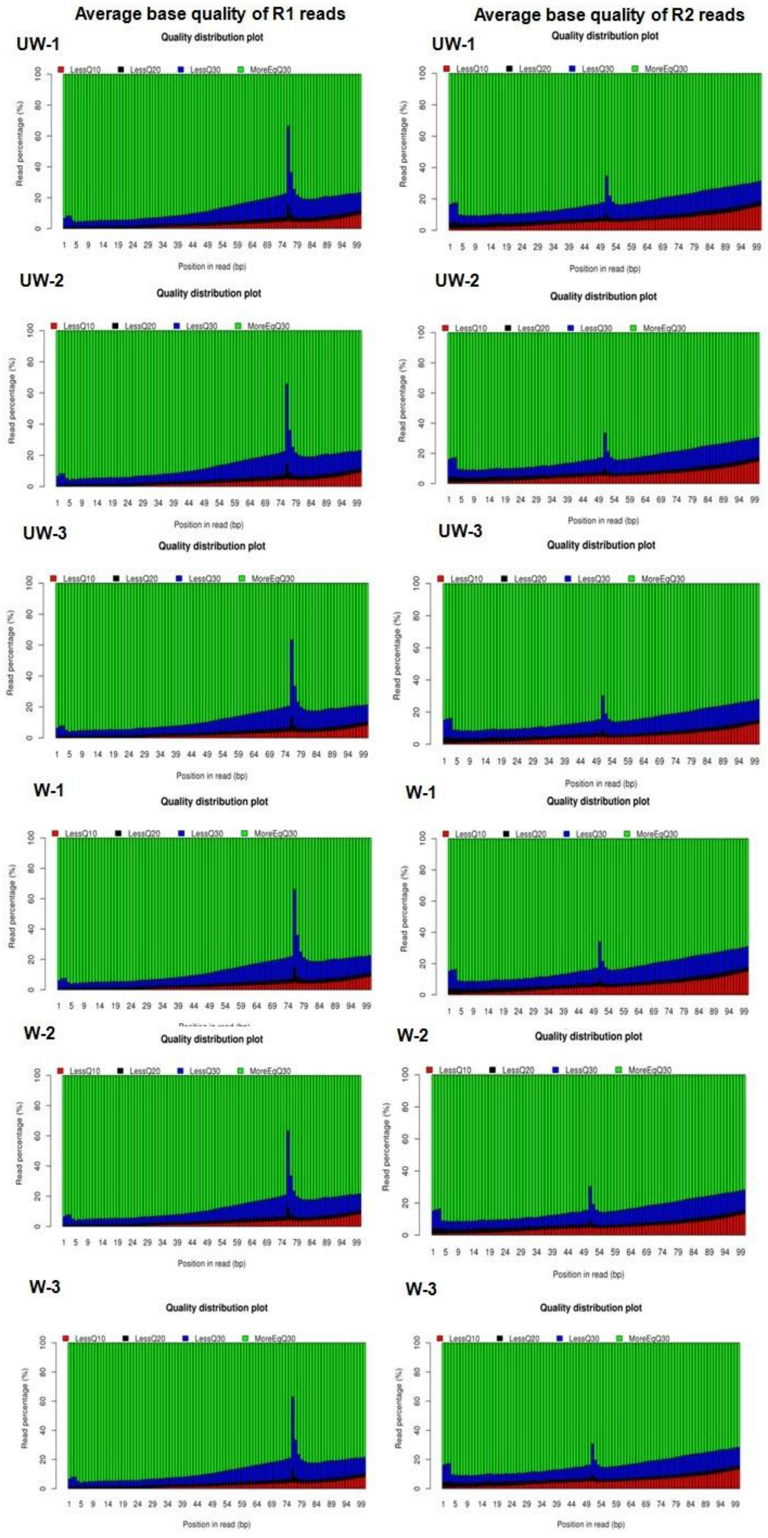

Figure S2: Scatter plot showing the variation in FPKM values among biological replicates. (A) Biological replicates of unwounded samples (B) Biological replicates of wounded samples.

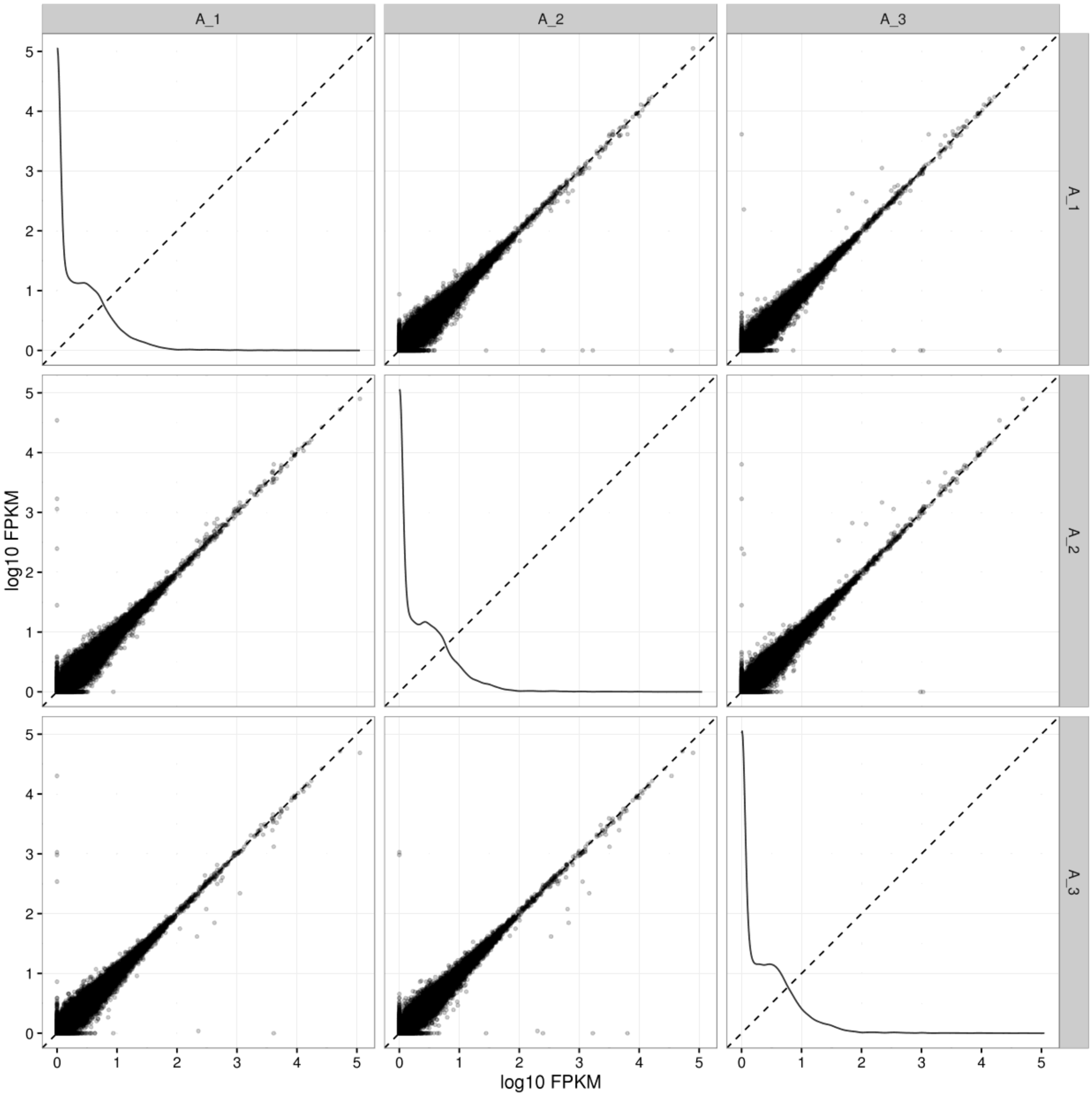

Figure S2 A

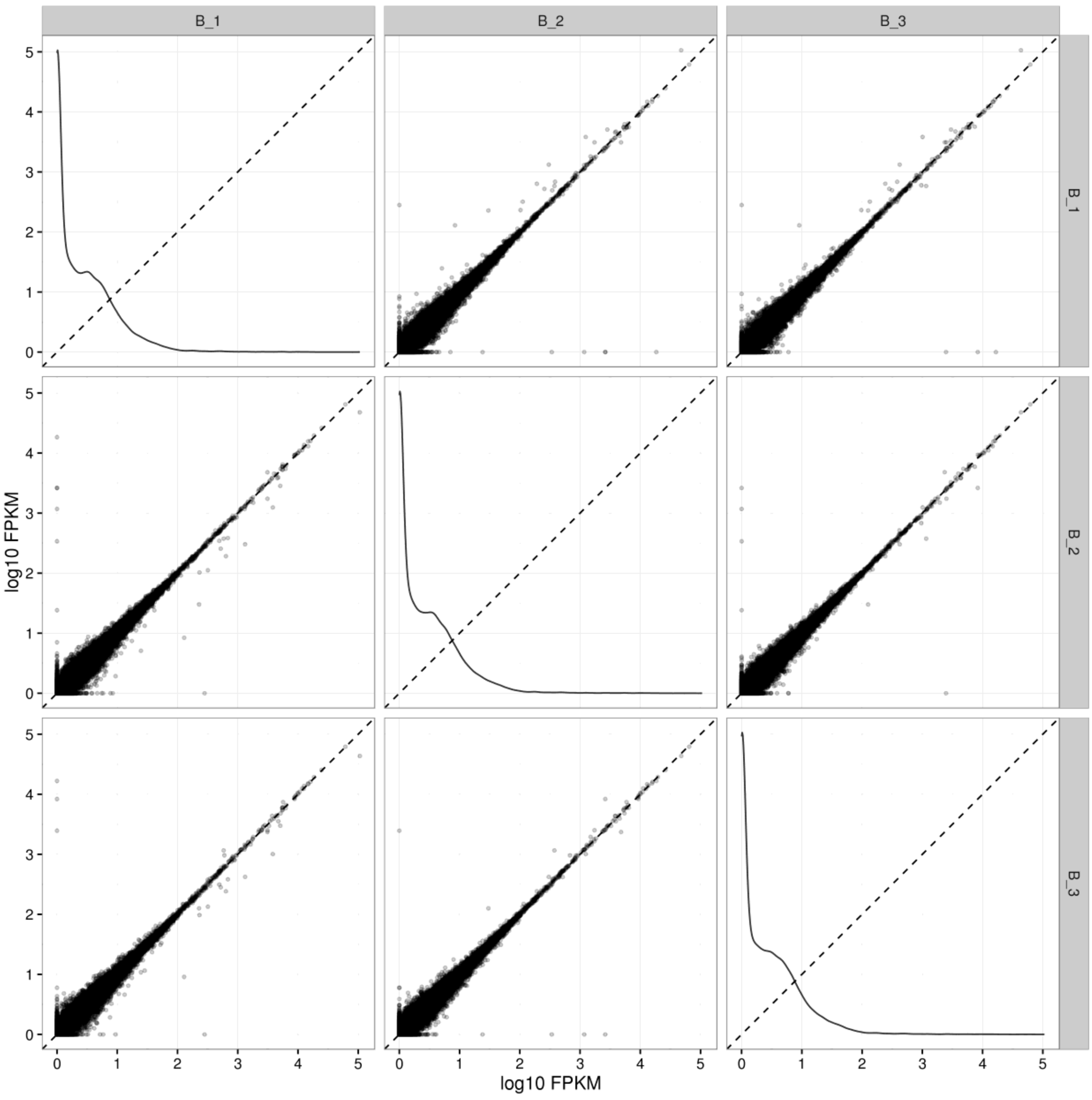

Figure S2 B

Figure S3: Histogram of Kyoto Encyclopedia of Genes and Genomes (KEGG) pathway enrichment analysis of DEGs (FDR < 0.05, $Log_2|FC| \leq -1$ and ≥ 1). The frequency of KEGG terms was analyzed with the help of KEGG Mapper (http://www.genome.jp/kegg/tool/map_pathway1.html) using the cam IDs retrieved from KEGG data base (http://www.genome.jp/kegg/). The x- and y-axes represent the names of clusters and the frequency of each cluster, respectively.

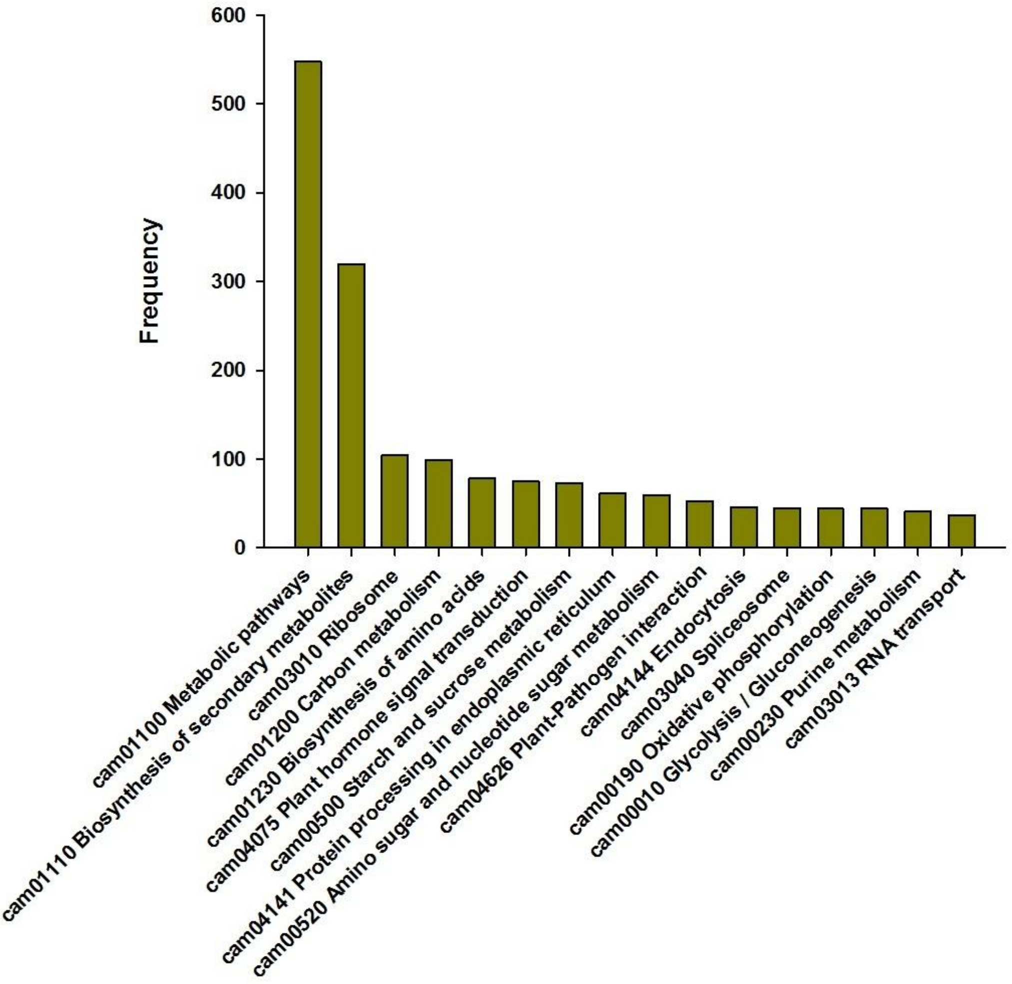

Figure S4: Heat map of expression of reference genes identified by Garg et al[34] between unwounded (UW) and wounded (W) chickpea leaf samples

AJ004960 = Elongation factor EF1α; GR406543 = Glutaredoxin protein;

FL512356 = Initiation factor 4a; AJ010224 = Glyceraldehyde-3-phosphate dehydrogenase;

GR405803 = Ubiquitin 5; GR398899 = Ubiquitin 10; GR406804 = Heat shock protein 90

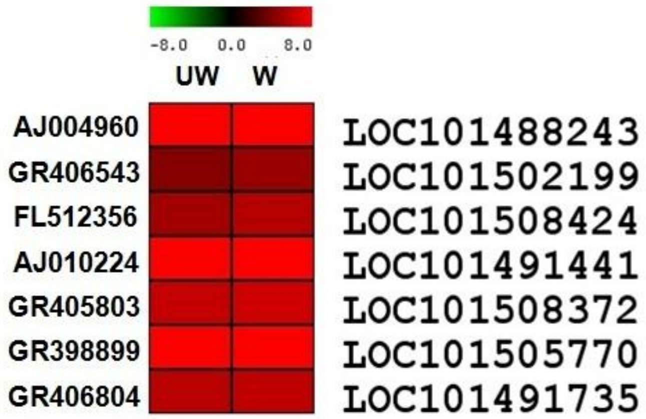

Table S1: Summary of the fastq file showing details of the sequencing data obtained (UW = unwounded, three replicates, W = 20 minute wounded, three replicates)

Sample Name	Total Reads	Clean Reads	Total Data (Gb)	GC (%)	% data>=Q 30	Aligned Read Count (%)
UW-1	13,818,784	12,808,668	1.38	47.34	84.2	12,043,947 (94.03%)
UW-2	13,890,822	12,986,254	1.38	46.38	84.5	12,243,290 (94.28%)
UW-3	14,404,232	13,828,812	1.44	45.02	85.9	13,040,575 (94.30%)
W-1	16,984,870	15,282,226	1.69	47.35	84.7	14,434,111 (94.45%)
W-2	16,408,146	15,475,626	1.64	45.06	85.7	14,590,868 (94.28%)
W-3	16,323,834	15,415,542	1.63	44.62	85.4	14,519,414 (94.19%)

Table S2: Differential wound-regulated genes involved in defence responses.

Putative Function	Accession No.	log_2-Fold Change	(p- value)
**Pathogenesis-related genes**			
protein YLS9	LOC101500729	3.9	0.00005
proteinase inhibitor	LOC101496018	1.6	0.0042
proteinase inhibitor	LOC101501374	1.3	0.0425
protein NDR1-like	LOC101511480	1.7	0.00005
endochitinase PR4	LOC101496543	5.1	0.0001
chitinase-like protein	LOC101494837	1.6	0.00005
endochitinase PR4	LOC105852061	1.4	0.00035
chitinase-like protein	LOC101492276	0.9	0.00005
endoglucanase	LOC101510949	1.2	0.00005
glucan endo-1,2-β-glucosidase 8-like	LOC101503370	2.9	0.0001
glucan endo-1,2- β-glucosidase 8-like	LOC101504732	2.7	0.00005
thaumatin-like protein	LOC101499398	2.4	0.00005
thaumatin-like protein	LOC101495985	-1.0	0.00005
thaumatin-like protein	LOC101503912	0.7	0.0001
endoglucanase	LOC101515001	4.0	0.0026
chitin elicitor receptor kinase 1	LOC101500451	1.1	0.00005
wound-induced protein	LOC101502145	1.6	0.00005
TMV resistance protein	LOC101511404	-1.0	0.00005
TMV resistance protein	LOC101502635	3.6	0.00005
TMV resistance protein	LOC101491849	3.3	0.0005
nematode resistance protein-like HSPRO2	LOC101512092	3.1	0.00005
nematode resistance protein-like	LOC101496827	2.3	0.00005

HSPRO2			
putative disease resistance RPP13	LOC101505907	4.9	0.00005
putative disease resistance protein RGA4	LOC101490582	3.5	0.00005
enhanced disease resistance 2-like	LOC101489098	1.3	0.00005
phenylalanine ammonia-lyase	LOC101509831	1.3	0.0569
dirigent protein 2-like	LOC101491323	1.6	0.00005
ankyrin repeat-containing protein	LOC101499879	1.1	0.00005
**Phenylpropanoid pathway**			
phenylalanine ammonia-lyase 2	LOC101509831	1.3	0.0569
4-coumarate-CoA ligase-like 5	LOC101511768	2.3	0.00005
cinnamoyl-CoA reductase 1-like	LOC101496722	1.1	0.00005
chalcone synthase	LOC105852659	0.6	0.0156
cinnamyl alcohol dehydrogenase	LOC101514054	0.6	0.0569
leucoanthocyanidin dioxygenase	LOC101512288	4.9	0.01715
caffeoylshikimate esterase	LOC101496529	2.3	0.00005
caffeoyl-CoA O-methyltransferase	LOC101508155	2.0	0.00005
methylenetetrahydrofolate reductase 2-like	LOC101515209	1.2	0.00005
flavin-containing monooxygenase FMO	LOC101505076	2.1	0.00005
laccase-4-like	LOC101513932	4.2	0.0061
4-hydroxyphenylpyruvate dioxygenase	LOC101503806	1.8	0.00005
UDP-glycosyltransferase 73C2-like	LOC101510567	-1.3	0.00005
**Cytochrome P450**			
cytochrome P450 86A8	LOC101495684	3.6	0.00005
cytochrome P450 94C1	LOC101509106	5.9	0.02473
cytochrome P450 77A1	LOC105852450	2.5	0.00075
cytochrome P450 77A3	LOC101513914	1.6	0.00005
cytochrome P450 84A1	LOC101491312	1.0	0.0041
cytochrome P450 71A3	LOC101497769	- 1.8	0.00005
cytochrome P450 704C1	LOC101494461	- 1.7	0.00005
cytochrome P450 71A1-like	LOC101506523	-1.6	0.00005
cytochrome P450 71A3-like	LOC101498428	-1.6	0.00005
**Oxidative Stress**			
glutathione S-transferase	LOC101488943	2.3	0.00005
glutathione S-transferase	LOC101494404	1.1	0.0043
peroxidase 64	LOC101514325	2.5	0.0005
peroxidase 63	LOC101495715	2.6	0.0005
peroxidase P7-like	LOC101501223	4.5	0.0026
catalase-4	LOC101513499	-0.6	0.00005
polyamine oxidase 2	LOC101514322	1.2	0.00005
respiratory burst oxidase homolog protein A	LOC101511451	1.5	0.00005
respiratory burst oxidase homolog protein B	LOC101499771	1.8	0.00005
respiratory burst oxidase homolog	LOC101488328	1.5	0.00005

protein C			
respiratory burst oxidase homolog protein D	LOC101491892	- 0.5	0.0075
reticuline oxidase-like protein	LOC101507115	1.7	0.00005
L-ascorbate oxidase	LOC101506817	4.8	0.00005
L-ascorbate oxidase	LOC101515454	4.1	0.0026
long-chain-alcohol oxidase FAO2	LOC101495423	-1.4	0.00035
monocopper oxidase-like protein SKU5	LOC101490477	2.9	0.00005
ferric reduction oxidase	LOC101511261	-1.4	0.00005
**Cell wall associated**			
arabinogalactan protein	LOC101505246	6.0	0.0026
arabinogalactan protein	LOC101503769	6.0	0.00005
cellulose synthase	LOC101509896	5.4	0.00005
cellulose synthase	LOC101489000	3.3	0.00005
cellulose synthase	LOC101488214	-1.1	0.00005
expansin-like	LOC101514490	3.5	0.00005
pectate lyase 1	LOC101515337	2.1	0.00005
xyloglucan endotransglucosylase/hydrolase	LOC101489781	8.0	0.0001
xyloglucan endotransglucosylase/hydrolase	LOC101510893	5.4	0.00005
polygalacturonase	LOC101489140	2.9	0.00005
polygalacturonase	LOC101503329	1.3	0.0001
glycine-rich cell wall structural protein	LOC101502810	2.2	0.00005
putative cell wall protein	LOC101508692	4.8	0.00305
leucine-rich repeat extensin-like	LOC101488829	3.7	0.00295
leucine-rich repeat extensin-like	LOC101511941	2.9	0.00005
proline-rich extensin	LOC101495711	2.4	0.00005
pectinesterase inhibitor 25	LOC101506095	3.7	0.00005
pectinesterase	LOC101490578	2.9	0.00035
Germin like proteins	LOC101510581	3.7	0.0133
Germin like proteins	LOC101492536	1.7	0.00085
**Heat shock proteins**			
heat shock cognate 70 kDa protein	LOC101496061	2.3	0.00005
heat shock factor protein HSF30	LOC101510605	1.3	0.00005
heat shock factor-binding protein 1-like	LOC101498596	1.2	0.00005
small heat shock protein	LOC101493857	- 0.9	0.00005

Table S3: Differential wound-regulated genes encoding signaling and regulatory proteins

Putative Function	Accession No.	log_2-Fold Change	(p- value)
**Transcription Factors**			
**Zinc finger**			
zinc finger protein ZAT10	LOC101503529	5.3	0.00005
zinc finger C3H domain-containing protein	LOC101488929	3.4	0.00005
B-box zinc finger protein 25	LOC101506782	- 1.8	0.00005
zinc finger protein 8	LOC101514113	- 2.0	0.00005
B-box zinc finger protein 32	LOC101494931	- 2.4	0.0037
dof zinc finger protein DOF5.4-like	LOC101508706	- 3.2	0.00005
**MYB**			
myb-related protein Myb4-like	LOC101508022	3.7	0.00005
transcription factor MYB44-like	LOC101507248	2.9	0.00005
transcription factor MYB44-like	LOC101491174	2.6	0.00005
target of Myb protein 1-like	LOC101508883	2.3	0.00005
transcription factor MYB108-like	LOC101495438	1.9	0.0043
transcription factor MYB48-like	LOC101489458	-2.1	0.00005
transcription factor GAMYB-like	LOC101499054	-1.7	0.0005
transcription factor MYB1R1-like	LOC101511722	-1.6	0.00005
**ERF**			
ERF017-like	LOC101496258	6.9	0.00005
ERF017-like	LOC101494182	5.9	0.00005
ERF026-like	LOC101507496	5.2	0.00005
ERF027	LOC101511542	5.2	0.00005
ERF014	LOC101495993	5.1	0.00005
ERF060	LOC101502773	- 2.3	0.00005
RAP2-1	LOC101501500	- 1.7	0.00005
RAP2-7	LOC101497653	- 1.4	0.00005
**WRKY**			
WRKY transcription factor 40	LOC101512217	4.6	0.0001
WRKY transcription factor 41	LOC101502574	4.4	0.00005
WRKY transcription factor 33	LOC101509113	4.4	0.00095
WRKY transcription factor 33	LOC101511519	4.1	0.00005
WRKY transcription factor 53	LOC101499178	3.3	0.00005
WRKY transcription factor 46	LOC101503062	3.0	0.00005
**bHLH**			
transcription factor bHLH18-like	LOC101507557	6.0	0.00005
transcription factor bHLH19	LOC101497663	5.4	0.00005
transcription factor bHLH35	LOC101512768	5.1	0.00005
transcription factor bHLH13-like	LOC101493372	2.2	0.00005
transcription factor bHLH113	LOC101510287	1.2	0.00005

transcription factor bHLH14	LOC101508717	- 1.7	0.00005
transcription factor bHLH143-like	LOC101495613	- 1.3	0.00005
transcription factor bHLH147-like	LOC101497649	-1.0	0.00435
transcription factor MYC2-like	LOC101507137	3.2	0.00005
**NAC**			
NAC domain-containing protein 2-like	LOC101501929	3.8	0.00005
NAC domain-containing protein 90	LOC101497878	3.2	0.00005
NAC domain-containing protein 67	LOC101514169	3.0	0.00005
NAC domain-containing protein 2-like	LOC101505565	1.9	0.00005
NAC transcription factor 29-like	LOC101509428	- 2.5	0.00005
NAC transcription factor 25-like	LOC101506350	- 1.7	0.00005
NAC transcription factor 29	LOC101499961	- 1.5	0.00615
**ARF**			
auxin response factor 8	LOC101496441	1.1	0.00005
auxin response factor 19	LOC101489666	- 0.9	0.00005
**MADS**			
MADS-box protein AGL24-like	LOC101503022	2.7	0.0012
MADS-box transcription factor 17	LOC101488623	- 0.9	0.00005
**GATA**			
GATA transcription factor 8	LOC101513365	3.6	0.00005
GATA transcription factor 7	LOC101509846	3.3	0.00005
GATA transcription factor 16-like	LOC101509226	1.1	0.0193
GATA transcription factor 22	LOC101504919	- 1.7	0.00005
GATA transcription factor 21	LOC101491168	- 1.6	0.00005
**Others**			
transcription factor PosF21	LOC101490377	3.1	0.00005
transcription factor DIVARICATA	LOC101511974	3.0	0.0002
transcription factor TCP7-like	LOC101492981	2.6	0.00005
transcription factor GTE7-like	LOC101502682	2.3	0.00005
transcription factor TCP7-like	LOC101493313	1.9	0.00005
transcription factor GLK1	LOC101513837	- 1.7	0.00005
transcription factor DIVARICATA	LOC101505171	- 1.5	0.00005
transcription factor LHW-like	LOC101506222	-1.5	0.00005
transcription factor EMB1444-like	LOC101511958	- 1.3	0.00005
homeobox-leucine zipper protein ATHB-6	LOC101514480	-1.2	0.00005
FAR1-RELATED SEQUENCE 10	LOC101488220	-1.1	
**Kinases/Phosphatases**			
**MAP kinase**			
MAPKKK	LOC101514482	3.3	0.00005
MPK-3	DQ659098	4.5	0.00005
MMK2-like	LOC101491525	2.7	0.00005
MAP- NTF6	LOC101496681	1.2	0.00005
mitogen-activated protein kinase 15-like	LOC101514058	- 0.7	0.00005
**Leucin rich repeat kinase**			

LRR receptor-like protein kinase	LOC101505704	5.3	0.00005
LRR receptor protein kinase MSP1-like	LOC101491649	3.3	0.00005
LRR receptor serine/threonine-protein kinase	LOC101509434	2.9	0.00005
LRR serine/threonine-protein kinase	LOC101507418	3.5	0.00005
LRR receptor-like protein kinase PXC1	LOC101494569	2.3	0.00155
leucine-rich repeat receptor-like	LOC101501880	- 1.2	0.00005
LRR serine/threonine-protein kinase BAM2	LOC101489895	- 1.0	0.00025
LRR receptor serine/threonine-protein kinase	LOC101492613	-1.4	0.00005
**CDPK**			
calcium-dependent protein kinase 28	LOC101502305	3.1	0.00005
calcium-dependent protein kinase 26-like	LOC101505073	3.0	0.00005
calcium-dependent protein kinase 2	LOC101511771	2.3	0.00005
calcium-dependent protein kinase SK5	LOC101512548	1.3	0.003
**G/L-type lectin**			
G-type lectin S-receptor-like ser/thr kinase	LOC101500928	2.8	0.00005
G-type lectin S-receptor-like ser/thr kinase	LOC101491401	2.6	0.00005
G-type lectin S-receptor-like ser/thr kinase	LOC101509945	2.1	0.00015
G-type lectin S-receptor-like ser/thr kinase	LOC101512757	1.6	0.00005
G-type lectin S-receptor-like ser/thr kinase	LOC101506092	1.5	0.00005
G-type lectin S-receptor-like ser/thr kinase	LOC101498183	1.5	0.00005
G-type lectin S-receptor-like ser/thr kinase	LOC101514619	- 1.1	0.00005
L-type lectin S-receptor-like ser/thr kinase	LOC101493160	1.7	0.0006
L-type lectin S-receptor-like ser/thr kinase	LOC101509371	1.5	0.00005
**CBL interacting protein**			
CBL-interacting ser/thr-protein kinase 9	LOC101507462	1.6	0.00005
CBL-interacting ser/thr-protein kinase 25	LOC101496895	2.3	0.00005
CBL-interacting ser/thr-protein kinase 25	LOC101510050	- 2.2	0.00005
CBL-interacting ser/thr-protein kinase 6	LOC101511702	- 1.0	0.00005
**phosphatidylinositol gamma 4-like**			
phosphatidylinositol 4-kinase gamma 4-like	LOC101509818	2.4	0.00005
phosphatidylinositol 4-kinase gamma 5	LOC101507936	2.0	0.00005
phosphatidylinositol 4-kinase gamma 5	LOC101512782	1.7	0.00005
phosphatidylinositol 4-kinase gamma 7	LOC101503810	-1.5	0.00005
**serine/threonine protein**			
ser/thr-protein kinase-like protein CCR4	LOC101508895	5.7	0.00005
ser/thr-protein kinase-like protein	LOC101495842	4.2	0.00005
ser/thr-protein kinase-like protein	LOC101497114	2.6	0.00005
serine/threonine-protein kinase TNNI3K	LOC101491445	2.6	0.00005
ser/thr-protein kinase-like protein	LOC101511372	2.5	0.00005
ser/thr-protein kinase-like protein CCR3	LOC101500234	2.3	0.00005
probable ser/thr-protein kinase DDB	LOC101492788	2.3	0.00005
ser/thr-protein kinase-like protein	LOC101490779	2.3	0.00005
ser/thr-protein kinase-like protein OXI1	LOC101509786	2.1	0.00045

ser/thr-protein kinase-like protein WNK5	LOC101506224	- 1.8	0.00005
ser/thr-protein kinase-like protein HT1	LOC101488226	- 1.1	0.0001
ser/thr-protein kinase-like protein WNK3	LOC101493627	- 1.1	0.00005
**proline-rich receptor-like**			
proline-rich receptor-like PERK1	LOC101491738	2.3	0.00005
proline-rich receptor-like PERK14	LOC101493861	-1.2	0.00005
**cysteine-rich receptor-like**			
cysteine-rich receptor-like protein kinase	LOC101511391	1.6	0.00005
cysteine-rich receptor-like protein kinase 10	LOC101497575	-1.6	0.00005
cysteine-rich receptor-like protein kinase 25	LOC101503576	-1.1	0.00005
**Others**			
chitin elicitor receptor kinase 1	LOC101500451	1.1	0.00005
death-associated protein kinase dapk-1	LOC105851564	1.5	0.00005
protein kinase byr2	LOC101508722	3.6	0.00005
wall-associated receptor kinase-like 14	LOC101493575	1.9	0.00005
wall-associated receptor kinase-like 14	LOC101510009	1.0	0.00055
**Phosphatases**			
acid phosphatase 1-like	LOC101503023	2.3	0.00005
protein phosphatase 2C 34	LOC101515600	-1.1	0.00015
protein phosphatase 2C 40	LOC101488562	-1.1	0.00055
protein phosphatase 2C 25	LOC101508412	6.8	0.00005
protein phosphatase 2C 39	LOC101498795	2.3	0.00005
protein phosphatase 2A	LOC101504593	1.3	0.00005
protein phosphatase 2A	LOC101489508	2.1	0.00005
**Epigenetic regulator**			
histone H2A.1	LOC101514392	3.4	0.00005
histone H4	LOC101513858	2.6	0.00005
histone H4	LOC101493881	2.1	0.00005
histone H4	LOC101491592	2.0	0.00005
histone H2B.3-like	LOC101502121	1.9	0.0041
probable histone H2B.1	LOC101506117	1.8	0.00005
probable histone H2A.3	LOC101490207	1.6	0.00005
histone-lysine N-methyltransferase	LOC101508428	1.2	0.00005
histone H1	LOC101498231	-1.6	0.00005
histone deacetylase	LOC101515579	-0.9	0.00005
**Receptors**			
B-cell receptor-associated protein 31	LOC101498526	1.0	0.0018
glutamate receptor 2.7-like	LOC101492454	2.2	0.00005
glutamate receptor 2.7-like	LOC101491994	0.6	0.00165
glutamate receptor 3.6	LOC101501975	-1.7	0.00005
glutamate receptor 3.4	LOC101505478	-0.6	0.0018
glutamate receptor 3.7	LOC101507003	-0.5	0.00005
glutamate receptor 2.7-like	LOC101491907	-0.5	0.0002
rhicadhesin receptor	LOC101505865	1.6	0.01785
systemin receptor SR160	LOC101509761	4.5	0.01785

toll/interleukin-1 receptor-like protein	LOC101496819	3.7	0.00345
**F-Box/E3-Ubiquitin ligase**			
**Tubby**			
tubby-like F-box protein	LOC101513372	3.1	0.00005
tubby-like F-box protein 8	LOC101494779	0.6	0.0001
**WD repeats**			
F-box/WD repeat-containing protein	LOC101511687	1.5	0.00005
F-box/WD-40 repeat-containing protein	LOC101509569	0.9	0.00005
WD repeat-containing protein 44-like	LOC101493225	-0.9	0.00005
WD repeat-containing protein 3	LOC101509269	-0.9	0.00005
**LRR**			
F-box/LRR-repeat protein 20-like	LOC101499815	-0.8	0.00005
putative F-box/FBD/LRR-repeat protein	LOC101505534	-0.9	0.0135
F-box/LRR-repeat protein	LOC101502550	-0.9	0.0041
F-box/LRR-repeat protein 4	LOC101496622	-0.9	0.001
**Kelch**			
F-box/kelch-repeat protein	LOC101514744	1.6	0.00005
F-box/kelch-repeat protein	LOC101508373	-1.5	0.00005
F-box/kelch-repeat protein	LOC101495912	-1.5	0.0001
F-box/kelch-repeat protein	LOC101511678	-1.6	0.00005
F-box/kelch-repeat protein	LOC101510735	-2.1	0.00005
F-box/kelch-repeat protein	LOC101495569	-2.3	0.00005
**F-box protein**			
F-box protein	LOC101506545	5.1	0.00045
probable F-box protein	LOC101506122	3.8	0.0026
F-box protein	LOC101507801	2.6	0.00005
F-box protein PP2-A12-like	LOC101504228	2.2	0.00005
putative F-box protein	LOC101507833	1.0	0.00005
F-box protein SKIP28	LOC101499399	-1.3	0.00005
F-box protein MAX2	LOC101508786	-1.4	0.00005
F-box protein PP2-A12-like	LOC101508855	-1.9	0.00005
F-box protein	LOC101491689	-2.7	0.00005
**RING**			
RING-H2 finger protein ATL40-like	LOC101507221	4.4	0.00005
RING-H2 finger protein ATL2-like	LOC101507635	4.3	0.00005
RING-H2 finger protein ATL3-like	LOC101505202	3.7	0.0039
RING-H2 finger protein ATL8	LOC101503467	-1.9	0.00005
RING-H2 finger protein ATL80-like	LOC101501916	-1.9	0.0047
RING-H2 finger protein ATL8-like	LOC101497131	-2.1	0.00005
**E3 ubiquitin-protein ligase**			
E3 ubiquitin-protein ligase SDIR1	LOC101496755	2.7	0.00005
E3 ubiquitin-protein ligase At1g63170-like	LOC101508240	-1.4	0.00005
E3 ubiquitin-protein ligase ATL6-like	LOC101509388	2.7	0.00005

E3 ubiquitin-protein ligase ATL6-like	LOC101503054	-1.8	0.00005
E3 ubiquitin-protein ligase MARCH11-like	LOC101500530	-1.2	0.00015
E3 ubiquitin-protein ligase MARCH1-like	LOC101498829	1.2	0.00075
E3 ubiquitin-protein ligase PUB23-like	LOC101503516	3.4	0.00055
E3 ubiquitin-protein ligase RGLG2-like	LOC101503410	1.5	0.00005
E3 ubiquitin-protein ligase RHA1B	LOC101491053	-2.1	0.00005
putative E3 ubiquitin-protein ligase XBAT34	LOC101507384	1.6	0.00005
probable E3 ubiquitin-protein ligase LUL4	LOC101497882	1.6	0.00005
E3 ubiquitin-protein ligase SIS3	LOC101492805	0.8	0.0018

Table S4: Differential wound-regulated genes related to hormonal responses.

**Putative Function**	**Accession No.**	**log_2-Fold Change**	**(p- value)**
**Jasmonic Acid**			
Linoleate 9S-lipoxygenase	LOC101491931	3.9	0.0002
Linoleate 9S-lipoxygenase	LOC101491624	4.6	0.00005
Linoleate 13S-lipoxygenase	LOC101501374	2.1	0.0002
Linoleate 13S-lipoxygenase	LOC101512038	1.0	0.00005
Allene oxide synthase	LOC101496543	2.6	0.00005
Allene oxide cyclase	LOC101500382	4.1	0.00265
Allene oxide cyclase	LOC101504350	1.9	0.00005
12-oxophytodienoate reductase	LOC101491096	- 0.5	0.00065
Peroxisomal acyl-coenzyme A oxidase 1	LOC101501296	1.0	0.00005
jasmonate O-methyltransferase	LOC101514869	- 0.7	0.0002
Jasmnate ZIM domain	LOC101488350	5.6	0.00035
Jasmnate ZIM domain	LOC101495051	4.0	0.00005
Jasmnate ZIM domain	LOC101491099	3.3	0.00005
NINJA (novel interactor of JAZ)	LOC101490870	- 0.7	0.0044
**Ethylene**			
S-adenosylmethionine synthase	LOC101502216	2.2	0.00005
S-adenosylmethionine synthase	LOC101511749	1.3	0.00005
1-aminocyclopropane-1-carboxylate synthase	LOC101514377	5.6	0.00005
1-aminocyclopropane-1-carboxylate oxidase	LOC101488870	- 0.6	0.00005
protein reversion-to-ethylene sensitivity1	LOC101508138	-1.4	0.0005

ethylene receptor	LOC101512933	-0.7	0.0001
ethylene insensitive	LOC101511446	-0.7	0.00005
ethylene-responsive transcription factor	LOC101512146	4.3	0.0007
ethylene-responsive transcription factor	LOC101494525	4.1	0.00005
**Salicylic Acid**			
phenylalanine ammonia-lyase	LOC101509831	1.3	0.0569
BAHD acyltransferase	LOC101495408	3.3	0.00005
regulatory protein NPR3	LOC101498401	1.2	0.00005
protein SUPPRESSOR OF npr1	LOC101497758	2.4	0.00005
protein ENHANCED DISEASE RESISTANCE	LOC101489098	1.3	0.0005
WRKY	LOC101512217	4.6	0.0001
WRKY	LOC101502574	4.4	0.00005
UGT74F1	LOC101497872	- 0.8	0.00035
**Abscisic Acid**			
zeaxanthin epoxidase	LOC101492490	- 1.4	0.00005
NCED	LOC101511615	- 1.4	0.00005
NCED	LOC101492033	2.5	0.00005
abscisic acid receptor PYL4	LOC101508615	6.4	0.00005
ABA- 8'-hydroxylase	LOC101499787	- 2.6	0.01475
ABA- 8'-hydroxylase	LOC101503447	- 1.1	0.0178
PP2C	LOC101508412	6.8	0.00005
ABSCISIC ACID-INSENSITIVE	LOC101503872	- 0.7	0.0011
**Auxin**			
transport inhibitor response	LOC101489568	- 0.9	0.00005
transport inhibitor response	LOC101503837	- 0.5	0.00005
auxin-binding protein	LOC101493286	4.0	0.00005
auxin response factor	LOC101489666	- 0.9	0.00005
auxin response factor	LOC101501408	- 0.8	0.00005
auxin response factor	LOC101496441	1.1	0.00005
auxin efflux carrier component	LOC101504769	- 1.0	0.00005
auxin efflux carrier component	LOC101515585	- 0.7	0.00005
auxin efflux carrier component	LOC101491826	1.6	0.00455
auxin-induced protein	LOC101509355	- 3.4	0.00005
auxin-induced protein	LOC101504605	- 1.6	0.0101
auxin-induced protein	LOC101514178	- 1.4	0.00005
auxin-induced protein	LOC101503373	1.0	0.00005
auxin-induced protein	LOC101514996	1.7	0.00005
auxin-induced protein	LOC101502357	2.0	0.0041
auxin-induced protein	LOC101495287	2.1	0.00215
auxin-induced protein	LOC101503044	5.6	0.00005
**Gibbrellic Acid**			
ent-copalyl diphosphate synthase	LOC101496179	- 1.1	0.00005
ent-kaurene oxidase	LOC101492682	- 0.9	0.0054

ent-kaurenoic acid oxidase	LOC101512107	- 1.4	0.00005
gibberellin 20 oxidase	LOC101491320	- 1.7	0.00005
gibberellin 2-beta-dioxygenase	LOC101494727	2.3	0.00005
gibberellin 2-beta-dioxygenase	LOC101515302	- 1.4	0.00005
gibberellin-regulated protein	LOC101495554	- 1.5	0.00005
gibberellin receptor GID1C	LOC101513592	- 0.7	0.0027
transcription factor PIF4	LOC101488979	- 1.2	0.0569
transcription factor PIF1	LOC101512389	-1.1	0.00005
transcription factor PIF3	LOC101490374	-1.5	0.00005
DELLA protein	LOC101507839	1.1	0.00005
**Cytokinin**			
cytokinin dehydrogenase 6	LOC101493924	1.9	0.0003
cytokinin dehydrogenase 7	LOC101495126	-1.8	0.00005
adenine phosphoribosyltransferase	LOC101502360	0.7	0.0001
histidine kinase	LOC101511355	-0.7	0.00005
histidine kinase	LOC101507033	-2.6	0.00005
**Brassinosteroid**			
BES1/BZR1 homolog protein	LOC101488568	2.7	0.00005
BRI1 kinase inhibitor 1-like	LOC101490493	-1.5	0.0016
BRI1 kinase inhibitor 1	LOC101491149	1.8	0.00025
BRI1-associated receptor kinase 1	LOC101506449	0.50	0.0028

Table S5: List of eight differentially wound responsive genes chosen from RNA seq data for validation by qPCR (with and without insect saliva) with their fold expression changes as obtained in the transcriptome.

Genes selected for real time validation	Accession No.	Log_2 (Fold Change)	(FDR)
TIFY10A-like	LOC101488350	5.63	0.00029
Xyloglucan endotrans hydrolase	LOC101489781	8.09	0.00054
Protein phosphatase 2C	LOC101508412	6.85	0.00029
ABA receptor PYL-4 like	LOC101508615	6.40	0.00029
Serine/Arginine-rich splicing factor 4-like	LOC101491743	2.97	0.00029
Protein kinase Byr-2 like	LOC101508722	3.61	0.00029
Cell wall protein	LOC101508692	4.85	0.00029
Ethylene response factor	LOC101512146	4.38	0.00305

Table S6: List of genes and primers used for study

Genes	Primer 5′-3′
TIFY10A-like (LOC101488350)	Forward: TCCAAGGCAGGAACATCATTTG<br>Reverse: CCATGCACCGTCGAAACAA
Xyloglucan endotransglucosylase hydrolase (LOC101489781)	Forward: GTTCTGTGTCTGACGCTTCCG<br>Reverse: GCATTCTGGTGGTAAACCCTGTG
Protein phosphatase 2C (LOC101508412)	Forward: TGTCGCGGTGTTTGGAGGAT<br>Reverse: AGTCTCGGGCTCTGCTGTCACC
ABA receptor PYL-4 like (LOC101508615)	Forward: CAAACCATCAACGCCTCCG<br>Reverse: CGGCGACGACGTTACAGCT
Serine/Arginine-rich splicing factor 4-like (LOC101491743)	Forward: CCGTACTTTCACCCCGCTCTT<br>Reverse: AAGCGTTCGCGCGATTTT
Protien kinase byr-2 like (LOC101508722)	Forward: AACGCAACGAGGTGACGATTC<br>Reverse: ATCGGAGCCGGAGATGGA
Cell wall protein (LOC101508692)	Forward: GTTATGTTCCTGGTGGCGACG<br>Reverse: ATGGACGAACTGCTGGTGGA
Ethylene response factor (LOC101512146)	Forward: GGACTCCATACGCCGTCACTTG<br>Reverse: TCGCCCCAATTCTCACTCAG
EF1alpha	Forward: TCCACCACTTGGTCGTTTTG<br>Reverse: CTTAATGACACCGACAGCAACAG
HSP-90	Forward: GCAGCATGGCTGGTTACATGT<br>Reverse: TGATGGGATTCTCAGGGTTGA
